# Using technology to scale-up training and supervision of community health workers in the psychosocial management of perinatal depression: a non-inferiority, randomized controlled trial

**DOI:** 10.1017/gmh.2019.7

**Published:** 2019-05-16

**Authors:** Atif Rahman, Parveen Akhtar, Syed Usman Hamdani, Najia Atif, Huma Nazir, Iftikhar Uddin, Anum Nisar, Zille Huma, Joanna Maselko, Siham Sikander, Shamsa Zafar

**Affiliations:** 1 University of Liverpool, Liverpool, UK; 2 Human Development Research Foundation, Islamabad, Pakistan; 3 Bacha Khan Medical College, Mardan, Pakistan; 4 University of North Carolina at Chapel Hill, Chapel Hill, North Carolina, USA; 5 Health Services Academy, Islamabad, Pakistan; 6 Fazaia Medical College, Islamabad, Pakistan

**Keywords:** Low- and middle-income countries, perinatal depression, psychosocial intervention, technology-assisted training and supervision, Thinking Healthy Programme

## Abstract

**Background.:**

The Thinking Healthy Programme (THP) is an evidence-based psychological intervention endorsed by the World Health Organization, tailored for non-specialist health workers in low- and middle-income countries. However, training and supervision of large numbers of health workers is a major challenge for the scale-up of THP. We developed a ‘Technology-Assisted Cascaded Training and Supervision system’ (TACTS) for THP consisting of a training application and cascaded supervision delivered from a distance.

**Methods.:**

A single-blind, non-inferiority, randomized controlled trial was conducted in District Swat, a post-conflict area of North Pakistan. Eighty community health workers (called Lady Health Workers or LHWs) were randomly assigned to either TACTS or conventional face-to-face training and supervision by a specialist. Competence of LHWs in delivering THP post-training was assessed by independent observers rating a therapeutic session using a standardized measure, the ‘Enhancing Assessment of Common Therapeutic factors’ (ENACT), immediately post-training and after 3 months. ENACT uses a Likert scale to score an observed interaction on 18 dimensions, with a total score of 54, and a higher score indicating greater competence.

**Results.:**

Results indicated no significant differences between health workers trained using TACTS and supervised from distance *v.* those trained and supervised by a specialist face-to-face (*mean ENACT score M*  =  24.97, s.d.  =  5.95 *v*. *M* =  27.27, s.d.  =  5.60, *p*  =  0.079, 95% CI 4.87–0.27) and at 3 months follow-up assessment (*M*  =  44.48, s.d.  =  3.97 *v*. *M* =  43.63, s.d.  =  6.34, *p*  =  0.53, CI −1.88 to 3.59).

**Conclusions.:**

TACTS can provide a promising tool for training and supervision of front-line workers in areas where there is a shortage of specialist trainers and supervisors.

## Background

Depressive disorders are the leading contributor to the global burden of disease among women of child-bearing age (Vos *et al*. 2012). Rates of perinatal depression in low- and middle-income countries (LMICs) range from 18% to 25% (Fisher *et al*. 2012), while in Pakistan, rates of 28–38% have been reported (Rahman *et al*. 2003 ; Khan *et al*. 2015). Problems such as depression can have devastating effects on the whole family, especially children (Kastrup, 2006). Studies have demonstrated strong independent associations with pre-term birth (Dayan *et al*. 2002; Grote *et al*. 2010; Jarde *et al*. 2016), poor growth and cognitive development (Rahman *et al*. 2007; Halfon *et al*. 2014; Bennett *et al*. 2016), higher rates of diarrheal diseases (Rahman *et al*. 2007), early cessation of breastfeeding (Rahman *et al*. 2016*a*), and poor socio-emotional development (Herba *et al*. 2016). In countries like Pakistan with some of the worst reported rates of infant mortality and morbidity (UNICEF, 2018) and the vast majority of mothers with depression receiving no treatment, the condition is a public health priority.

Psychological interventions are the first line of treatment for depression. While most LMICs including Pakistan have vastly underdeveloped specialist facilities for mental health, a number of trials from LMICs show that non-specialists can deliver them effectively (Rahman *et al*. 2008, 2016*b*; Patel *et al*. 2010; Chibanda *et al*. 2016). The Thinking Healthy Programme (THP), developed in Pakistan, is a cognitive behavior therapy (CBT)-based intervention for perinatal depression, delivered by lay community health workers (CHWs). THP consists of 16 sessions, starting from the last trimester of pregnancy to 10th month postnatal. The intervention employs imagery techniques by using culturally appropriate illustrations/pictures to help women identify unhelpful thoughts, alternative ways of thinking (helpful thoughts), putting these helpful thoughts into action, and problem solving when issues arise in practicing new behaviors (Rahman *et al*. 2008, 2013). The THP is the first psychological intervention to be incorporated into the WHO's flagship Mental Health Gap Action Programme (mhGAP) (World Health Organization, 2016).

Despite these advances, the majority of women with perinatal depression in low-income countries do not receive the treatment and a key barrier is the extensive training, supervision, and monitoring required by non-specialists to ensure they deliver the complex intervention to fidelity. Training of a large number of health workers is not feasible, costly, time consuming, and difficult to arrange (Murray *et al*. 2014). Moreover, ensuring the quality and consistency in training and supervision at scale can be challenging (Mangham & Hanson, 2010).

The recent Lancet Commission on Global Mental Health (Patel *et al*. 2018) has highlighted the use of digital technology as a major area for future research to assist the scale-up of mental health interventions. In recent years, digital mental health technologies such as web-based platforms and mobile applications have been frequently cited as potential methods of extending evidence-based interventions (Naslund *et al*. 2017). In Pakistan, 87% of households own a mobile phone (National Institute of Population Studies, 2013), indicating the potential of digital technology for health promotion. However, at present, there are no Applications (Apps) that can assist in training a CHW to deliver an evidence-based intervention effectively in low-income settings (Fairburn & Cooper, 2011, Fairburn *et al.* 2017). Additionally, few studies have employed rigorous methodologies to evaluate the technological solutions to scaled-up training.

We developed and tested a technology-assisted training and supervision system for CHWs to be trained in an evidence-based intervention for perinatal depression in a post-conflict area of Pakistan to establish whether it can be an alternative to conventional specialist-led face-to-face training and supervision.

## Methods

### Study design

A single-blind, non-inferiority, individual randomized controlled trial design was employed. The non-inferiority design was chosen because a novel method of training was being compared with an established standard method of training.

### Settings and participants

The study was conducted in District Swat, Khyber Pakhtunkhwa province, in the north of Pakistan. Swat has been exposed to multiple humanitarian crises over the last decade including large-scale armed conflict and floods. Following an insurgency by armed militants in 2006–2009, a massive military operation was carried out to regain control of the district. Around 2.5 million people were internally displaced as a result of the conflict between militants and the army in 2007 (Bile & Hafeez, 2009). While the conflict continued, devastating floods in 2010 resulted in thousands of people losing their homes and causing destruction to roads, schools, and health facilities. Health systems were seriously affected. Almost one-third of the health facilities were destroyed (Din *et al*. 2012). Currently, the health systems are fragile and transitioning toward normalcy. The psychological sequelae of these humanitarian disasters are apparent even years later; an epidemiological study reporting 38% of pregnant women had clinically significant psychological distress (Khan *et al*. 2015).

**Fig. 1.**
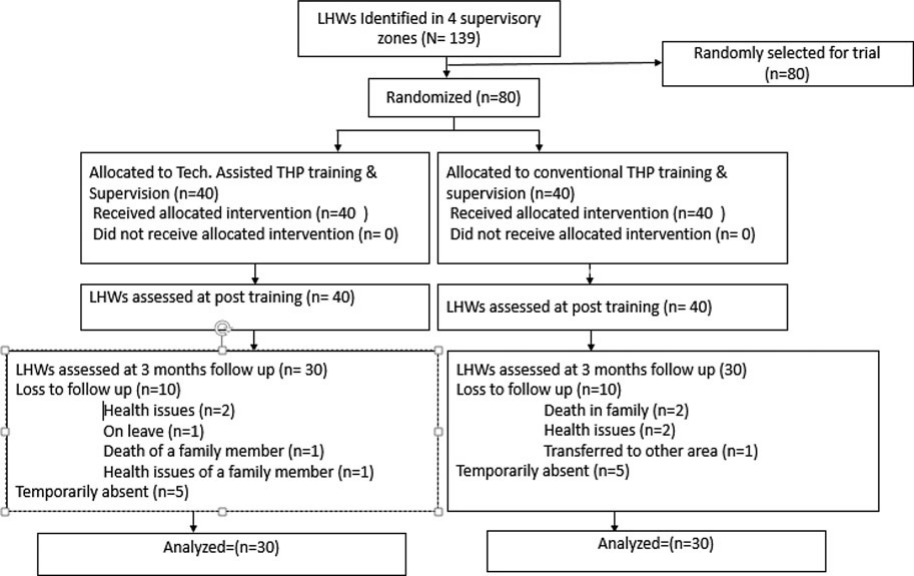
Participants' flow.

**Fig. 2.**
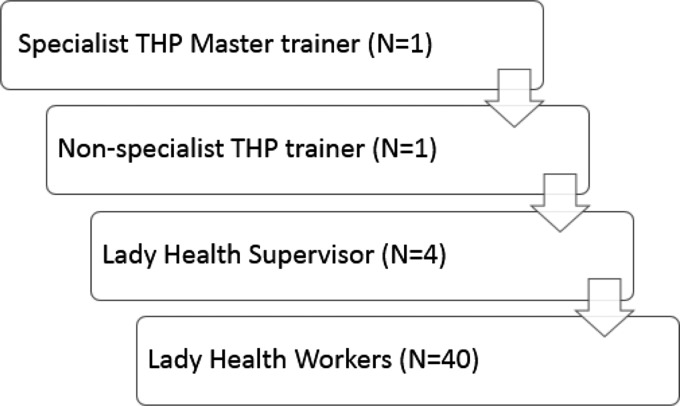
Cascaded training and supervision model in TACTS.

In rural Pakistan, the community-based maternal and child health care is delivered through CHWs called Lady Health Workers (LHWs). LHWs are local women employed by Primary Health Care (PHC) under the National Programme for Family Planning and PHC initiated in 1994. LHWs are trained and supervised by Lady Health Supervisors (LHSs). Each LHS, based at the PHC facility, supervises between 15 and 20 LHWs. LHSs and LHWs receive no training to provide mental health interventions. The current study was conducted from March 2016 to November 2016 in three peri-urban Union Councils of Swat: Faizabad, Rangmohalla, and Saidu Sharif (a Union Council is the smallest administrative unit within a district). To recruit participants, the LHWs program administration in the three Union Councils was approached and information about the study provided. The LHWs program was requested to provide lists of LHSs and LHWs working in the Union Councils. All the LHSs and LHWs in the list were informed about the study. From the list of 139 LHWs provided by LHW program, 80 LHWs were randomly selected. Figure 1 Figure 2 shows the flow of participants in the study.

The study was approved by the Ethics Review Committee of the Human Development Research Foundation. All participants provided written informed consent to participate in the trial. Permission was taken from women whose households were visited for observations of routinely delivered sessions. The full trial protocol has been published previously (Zafar *et al*. 2016).

### Technology-Assisted Cascaded Training and Supervision delivered to the intervention group

We adapted the original Urdu language paper version of the THP to a Technology-Assisted Cascade Training and Supervision (TACTS) system that included: (a) tablet-based application allowing standardized training to be delivered by non-specialist trainers; and (b) a cascade training/supervision model (Figure 2) where a specialist THP master trainer trained non-specialist THP trainers, who in turn trained and supervised LHSs. These LHSs then cascaded the training to the LHWs by integrating it into their routine training and supervision schedule. This cascaded model of training and supervision has been described as a feasible way of building capacity in mental health interventions at large-scale in LMICs (Murray *et al*. 2011).

Building on our previous work in this area (Hamdani *et al*. 2015), we used a multimedia android-based training Application. Training materials were converted into narrative scripts in the Urdu language by a panel of THP trainers. Culturally appropriate real-life characters representing the trainers and the trainees were developed. An artist converted the characters into ‘Avatars’ (i.e. graphic images representing each character in the narrative), which were used to voice the narrative scripts. The narratives, with individual avatars representing LHWs, mothers, and key family members, were demonstrated through fictional scenarios depicting skills such as effective use of counseling, collaboration with the mothers' families, guided discovery using pictures (i.e. a style of questioning to probe mother's health beliefs), and setting health-related tasks. To enhance the learning of THP delivery skills, an option to view short videos of role plays was provided. The entire training process was interactive. The software was designed to prompt trainees to be involved in interactive activities such as commenting on the role plays, reflection on their learning, sharing of relevant experiences, and brain-storming about problem-solving strategies. These activities were designed to mimic activities conducted during face-to-face specialist-led training.

In the TACTS arm, a non-specialist THP trainer (psychology graduate, trained by specialist THP master trainer in a 5-day workshop) delivered the 20 h technology-assisted training spread over 5 days to the LHSs using the TACTS system. The LHSs then cascaded the 5-day training using the same TACTS system to 40 LHWs. The main role of the LHS facilitator was to help the LHWs navigate the App, stimulate discussion, and organize the role plays.

#### Supervision

The LHSs supervised the LHWs using TACTS as part of their routine monthly group supervisions at Basic Health Units. Supervision was focused on, sharing experiences to enhance motivation and problem solve as a group, rehearsing core intervention concepts via role plays and re-watching the training videos. Supervision was an integral part of promoting experiential learning following the training, and a separate module on supervision was developed for the LHSs to integrate this in their routine monthly group supervision of LHWs. This module consisted of guidelines for revising core intervention elements via role plays, reviewing the work of LHWs (case load, sessions delivered, difficulties encountered, and adverse events), sharing experiences, problem solving, and motivating LHWs.

LHSs were supervised by the non-specialist THP trainer remotely via Skype in a monthly group supervision of 2 h. LHSs discussed the challenges they faced during supervision of LHWs and difficulties they experienced in providing support and feedback to LHWs, addressed motivation and work stress, and reinforced intervention core concepts.

The non-specialist THP trainer received monthly supervision by a specialist THP master trainer for 1 h via Skype. Supervision focused on difficulties experienced in providing support and feedback to LHSs.

### Conventional face-to-face training and supervision delivered to the control group

The LHWs in the control group were trained directly by specialist THP master trainers in a 5-day training program, using THP training materials (THP training manual and job aid). The specialist THP master trainers were mental health specialists – psychologists trained in CBT with an in-depth understanding of THP. During the training, trainers explained the core concepts of the intervention. Role plays were conducted to enhance LHWs' skills in counseling, family engagement, and managing challenging situations. Training was a combination of lectures, group discussions, role plays, and feedback on the role plays by the trainers and peers.

#### Supervision

Specialist THP master trainers provided monthly face-to-face group supervision directly to the LHWs. The average duration of a supportive supervision session was 2 h. Supervision focused on positive as well as challenging experiences of LHWs and brainstorming solutions as a group. Motivation of LHWs was ensured by sharing of success stories. Intervention content was rehearsed via role play followed by feedback from the peers and trainers.

The LHWs in both arms delivered the intervention to women in the community using the original paper-based THP manual.

### Measures

The primary outcome was the competence of LHWs at 3 months post-training, measured by the ENhancing Assessment of Common Therapeutic factors (ENACT) rating scale, developed by Kohrt *et al*. (2015). ENACT is an 18-item scale to assess the competence of non-specialists via role plays or direct observation of a therapy session. The items are listed in Table 1. ENACT has been developed using a rigorous methodology and has shown good psychometric properties. Each item (also called a domain) is scored on a scale from 1 to 3, where 1  = needs improvement, 2  = partially done, 3  = done well. A composite score can be computed by adding all the items. The authors recommend that following training and practice sessions under supervision, a score of 80% of the total possible score represents a satisfactory level of competence. For the present study, an adapted ENACT composed of 16 items was used (excluding items 17 and 18 as these were more clinical relating to confidentiality and risk management). A score of 38 indicated the 80% level of satisfactory competence. ENACT has been used in Pakistan previously to measure the competence of health workers (Sikander *et al*. 2015).

**Table 1.** Enhancing Assessment of Common Therapeutic factors (ENACT) domains and items (adapted for the Thinking Healthy Programme)

**Item 1: Developing a bond without verbal communication and listening empathically: eye contact, facial expressions, and body language** **1 = Needs improvement **= no eye contact with the mother or staring constantly, expressing anger, mocking at her, interrupting the mother repeatedly, ignoring her, talking on the phone without asking for permission **2 = Done partially **= showing consistent lack of interest through body language, rare eye contact, not expressing emotions, presence **3 = Done well **= proper eye contact with the mother during conversation/discussion, smiling according to situation, proper seating arrangement and showing interest by leaning towards mother, and listening attentively using hmm…, yes, or any other local expression
**Item 2: Communication skills: asking open-ended questions, summarizing, and explaining the discussion** **1 = Needs improvement **= asking close-ended questions, for example, will you do this? Can you do this? **2 = Done partially **= asking open-ended questions but not going in detail and not asking for mother's opinion after summarizing the discussion **3 = Done well **= asking open-ended questions, summarizing the discussion and asking the details, for example, what happened? Tell me more
**Item 3: Developing a relationship (when the other person feels comfortable talking to you) and introducing yourself** **1 = Needs improvement **= Lady Health Worker does not introduce herself or make the mother feel comfortable or LHW talks mostly about her personal experiences during the session **2 = Done partially **= Lady Health Worker introduces herself but does not make the conversation comfortable for the mother by chit chat or the LHW talks about herself to the mother but it has no link with mother's situation **3 = Done well **= Lady Health Worker introduces herself, tries to make the conversation easy for the mother, and shares her personal experiences relating to the mother's situation/condition
**Item 4: In-depth understanding of the matter, explaining, and telling her that such emotions can be felt often** **1 = Needs improvement **= Lady Health Worker does not talk about mothers feelings or makes her own judgment or criticizes mother's feelings (e.g. you should not think that, you should stop thinking and feeling about it) **2 = Done partially **= LHW asks about mother's feelings and emotions but does not empathize with her, agree with her feelings, or ask for details **3 = Done well **= LHW explains the mother's feelings by relating them to the current situations and if appropriate, tells her that such emotions can be felt during these situations
**Item 5: Dealing with empathy, warmth, and sincerity (with being pretentious)** **1 = Needs improvement **= Lady Health Worker criticizes mother's concerns and complaints or behaves angrily or rejects her views **2 = Done partially **= Generally, Lady Health Worker's attitude is warm and friendly but she does not have the ability to empathize with her/relate with mother's perspective **3 = Done well **= Lady Health Worker expresses that she understands that the mother feels exactly how mostly people feel during this situation
**Item 6: Viewing daily activities and effects on life** **1 = Needs improvement **= Lady Health Worker does not ask the mother about her thoughts, feelings, and psychological issues and their effects on her life **2 = Done partially **= Lady Health Worker asks about daily activities and tasks but does not link it to mental or psychological problems **3 = Done well **= Lady Health Worker talks about the connection between psychological problems and daily activities
**Item 7: Knowing about what explanations (simple/common and explanatory model) do the mother and her social support (family members and friends) give about mother**'**s problems** **1 = Needs improvement **= Lady Health Worker does not ask the mother about reasons of her problems, or she makes her own judgment, criticizes mother when she gives any explanation (e.g. evil eye or black magic are not the cause of your problems, this is an ignorant and orthodox way of thinking) **2 = Done partially **= Lady Health Worker asks the mother about reasons for her problems but does not probe further if her family members also think the same way? (e.g. when in introductory session two sides of a picture are shown to know the perception of family members) **3 = Done well = **Lady Health Worker asks the mother about the reason and asks if her family/people in her social circle describe it the same way or differently
**Item 8: Method of dealing with problems and using the pre-existing methods of problem solving** **1 = Needs improvement **= Lady Health Worker does not ask the mother about how she deals with her problems or makes her own opinion about it (for instance, why do you think it is beneficial or not) **2 = Done partially = **Lady Health Worker talks about dealing with problems and already existing solution but does not tell in a positive manner **3 = Done well = **Lady Health Worker asks the mother about methods to deal with difficulties and then explains in a positive way
**Item 9: Reviewing the mother**'**s ongoing life incidents and circumstances and drawing their link with the mother**'**s psychological and social satisfaction** **1 = Needs improvement **= Lady Health Worker does not talk about incidents which create problems **2 = Done partially **= Lady Health Worker talks about life's incidents and circumstances/issues but does not connect the effects of unhealthy thoughts and moods with mother and child's satisfaction (for instance, when LHW shows pictures A, B, and C during the session) **3 = Done well **= Lady Health Worker talks about life incidents and circumstances and connects the effects of unhealthy thoughts and moods with mother and child's satisfaction (e.g. when she shows pictures D, E, and F during the session)
**Item10: Reviewing the problems of mother**'**s own health** **1 = Needs improvement = ** LHW does not talk about the thoughts/concerns of the mother about the mother's personal health **2 = Done partially = ** LHW talks about the thoughts/concerns of the mother about her personal health but does not draw the link between the unhealthy thoughts, mood, behaviors, and their effects (with the help of picture and examples) **3 = Done well **= LHW talks about the mother's thought/concerns regarding her personal health and wherever it is needed she draws a detailed link between the unhealthy thoughts, mood, behaviors, and their effects (with the help of pictures and examples)
**Item 11: Appropriate involvement of family members and other care takers** **1 = Needs improvement = **when any family member is present: During the session, LHW ignores the family or only talks to the family members and ignores the mother. When no family member is present: LHW does not talk about the family at all **2 = Done partially = **when family members are present: LHW talks to both, the mother and the child, but does not help the mother and the family during the session to communicate with each other. When the family member is not present: LHW talks about the family involvement but does not take the mother's view if she wants to involve the family or not **3 = Done well** **= **when any family member is present: LHW encourages and helps in the communication between the mother and the family member. When the family member is not present: LHW asks mother about family involvement and guides her
**Item 12: Setting the goals mutually and talking about the mother**'**s expectations** **1 = Needs improvement = **LHW does not ask the mother about her goals and expectations regarding the treatment, or LHW just tells the mother what to do without asking about her expectations **2 = Done partially = **LHW talks to mother about the goal/aim but does not discuss if this goal/aim is achievable **3 = Done well** **= **LHW talks to the mother about the goal that what is achievable through treatment and what is not, and mother and LHW mutually decide the method/procedure of treatment
**Item 13: giving hope for achievable change** **1 = Needs improvement **= LHW either does not give any hope (i.e. you will never get well) or gives unrealistic hopes about the treatment and the betterment through it (i.e. you will get well within few weeks and there will be no issues after that/in future) **2 = Done partially **= LHW ambiguously tells the mother about what will happen during the treatment **3 = Done well **= LHW makes/helps the mother to feel positive about the future and gives her achievable hopes about what can and cannot be achieved through the treatment. LHW analyzes the mother's understanding of achievable change
**Item14: Talking about mental health according to the level of understanding of local people** **1 = Needs Improvement** = LHW uses complicated or embarrassing words while talking about mental health or she does not explain how the treatment would work **2 = Done partially **= LHW rarely uses any complicated words and does not use embarrassing words but she is unable to make the mother or the local people understand about the mother's mental health **3 = Done well **= LHW uses local proverbs and non-embarrassing language, according to the level of understanding of mother and local people, to talk about mental health and makes sure that the mother understands
**Item 15: Steps for problem solving: 1^st^ step: guiding how to recognize the unhealthy thinking; 2^nd^ step: guiding how to replace the unhealthy thoughts with healthy thoughts; 3^rd^ step: exercising and adopting the healthy thinking** **1 = Needs improvement = ** LHW works with the mother to identify her unhealthy thinking **2 = Done partially **= LHW helps the mother to identify the unhealthy thoughts and changing it into healthy thinking **3 = Done well **= LHW helps the mother (1) to identify the unhealthy thoughts, (2) to change the unhealthy thoughts into healthy thoughts, (3) to decide about the work to be done for adopting the healthy thinking according to the health chart
**Item 16: Asking about (mother**'**s) opinion when a suggestion or advice is given while deciding the task** **1 = Needs improvement **= LHW tells the mother what to do without asking what she (mother) wants or what is easy/doable for her, or does not give any suggestion at all **2 = Done partially = ** LHW explains to the mother in a focused manner, for example, tells her to sleep for 7 h at night while talking about rest chart but does not ask the mother if this suggestion is helpful for her **3 = Done well = ** When the mother asks, LHW gives some advice and then asks about the mother's opinion about the advice
**Item 17: Explaining and promoting (ensuring) privacy** **1 = Needs improvement **= LHW does not take care about privacy or does not talk according to the occasion **2 = Done partially **= LHW tells the mother that everything should be kept private but does not mention those things which could harm one's self or anyone else are exempted. LHW talked about private matters even while the session was not done privately **3 = Done well = ** LHW explains to the mother that this conversation should be kept private except for the things that could harm her or anyone else. LHW takes care of the fact that the session is private or not, and talks accordingly
**Item 18: Causing harm to one**'**s own self, causing harm to others, analyzing the harm caused by others, and mutually planning to deal with it** **1 = Needs improvement **= LHW does not ask about harming one's own self or anyone else **2 = Done partially **= LHW explains about causing harm to one's own self or others but does not help the mother in planning to deal with it **3 = Done well **= LHW talks about causing harm to one's own self or others and guides the mother for an appropriate strategy

Competency assessments were conducted immediately after training (post-training assessment) and at 3 months post-training (follow-up assessment). Post-training assessment was conducted using structured role plays, while follow-up assessment was conducted through live observation by an assessor blind to the allocation status of the LHWs.

In addition to competence, we collected data on the cost from a program perspective. Data were collected on (1) direct costs associated with training of LHWs in THP using the TACTS system, and (2) information on the costs associated with the training and support of LHWs in the THP by the specialists following the conventional model. Data were also collected on the opportunity costs associated with the specialists' time. The information was gathered through semi-structured interviews with trainers covering details such as the venue of the training (the training space used), and the average number of hours worked by the specialists, LHSs, THP trainers, and LHWs. Data were collected throughout the study period. Information was also collected on the cost of developing TACTS and other related costs, e.g. communication costs, logistics costs, training material, and stationary.

### Sample and power calculations

The primary outcome of the study was the mean competence scores immediately post-training and at 3 months. We defined non-inferiority as a difference of five points or less (corresponding to a 10% difference in the outcome measure score) in the mean competence score between the two groups. A sample size of 80 LHWs (40 LHWs in each arm) provided 99% power, accounting for an attrition rate of 25% at 3 months follow-up, to detect a five-point margin with a 0.05 one-sided *α* level.

### Randomization and masking

The unit of randomization was the LHW. In all, 160 LHWs within the three Union Councils were identified. We randomly allocated 80 LHWs on a 1:1 ratio, stratified on the basis of LHS (equal number of LHWs from each supervisory zone). Randomization was conducted by an independent, off-site team member using a computer software. Allocation concealment was ensured by keeping the random assignments in sequentially numbered, opaque, sealed envelopes at the off-site center. Only outcome assessors were blind to the allocation status.

### Data analysis

Quantitative data were analyzed using SPSS v21. Descriptive statistics (means and standard deviations) were computed for demographic characteristics. Mean differences in competence scores of two groups were computed using the independent sample *t* test.

## Results

Figure 1 presents the trial profile. The mean age of the participating LHWs was 35.33 years (s.d.  = 7.71) and the mean period of work experience was 12.15 (s.d.  = 6.26) years. No significant differences were observed in demographic characteristics between both arms (Table 2). All the participants completed the training and post-training assessment. At primary end-point (3 months follow-up), 30 LHWs (75%) completed the assessment.

**Table 2.** Demographic characteristics of LHWs

	TACTS (*n* = 40)	Specialist-led training (*n* = 40)
	M (s.d.)	M (s.d.)
Age	35.58 (6.53)	35.33 (7.71)
Work experience	12.94 (5.43)	12.15 (6.26)

Results indicated no significant differences in the mean ENACT scores of the intervention and control groups at post-training (*M*  =  24.97, s.d.  =  5.95 *v*. *M*  =  27.27, s.d.  =  5.60, *p*  =  0.079, CI −4.87 to 0.27). Competency scores in both groups improved at 3 months follow-up. However, no significant differences were observed in control and intervention arm scores at 3 months follow-up (*M*  =  44.48, s.d.  =  3.97 *v*. *M* =  43.63, s.d.  =  6.34, *p*  =  0.53, CI −1.88 to 3.59). The results are summarized in Table 3. Twenty-seven out of 30 (67.5%) LHWs in TACTS arm and 28 out of 30 (70%) LHWs in conventional arm achieved competence (score above 80%) at follow-up assessment.

**Table 3.** Mean differences in primary outcome scores (competence) at post-training and 3 months post-training

	*n*	*M* (s.d.)	*n*	*M* (s.d.)	Difference in means (95% CI)
Post-training	40	24.97 (5.95)	40	27.27(5.60)	2.3 (−4.87 to 0.27)
3 months follow-up	30	44.48 (3.97)	30	43.63 (6.34)	0.85 (−1.88 to 3.59)

### Training costs

We found that the cost of training and supervision was 17648 PKR (USD 170) in the conventional training arm and 12195 PKR (USD 117) in the TACTS arm per LHW^1^^†^. The technology-assisted training was about 30% less expensive than the specialist-led training and supervision, yet competence levels achieved were similar.

## Discussion

This study evaluated conventional specialist-delivered face-to-face training of an evidence-based intervention for perinatal depression *v.* technology-assisted training by routine supervisors to LHWs in a post-conflict area of Pakistan. The results showed that similar levels of competence were achieved in both arms at post-training and 3 months follow-up, while the costs of THP-TACTS were 30% less than the specialist-led training and supervision.

The competency of LHWs improved in both arms over time with practice under monthly supportive supervision. This indicates that experiential learning and supportive supervision are crucial for such interventions. This also indicates that training and supervision with TACTS was effective in improving LHWs' skills, without the need for a specialist supervisor. Considering the lack of mental health specialists in resource-poor settings, this cascaded training and supervision, integrated within the healthcare system, could be a potential way to ensure delivery of psychological interventions with quality. Moreover, TACTS was found to be cheaper than the conventional training.

Technologies have been used in training health workers for different health conditions in LMICs. These include the use of mobile phone-assisted training health of workers in care of HIV (Zolfo *et al*. 2010), identification of breast cancer (Alipour *et al*. 2014), antenatal (Palazuelos *et al*. 2013), and neonatal care (Lund *et al*. 2016). Few studies demonstrate the use and effectiveness of such technologies for training health workers in delivering mental health interventions, especially for a common mental disorder. One such example is the proof of concept study in the UK where Fairburn *et al*. (2017) conducted web-based 9 h CBT training of 102 therapists for eating disorders and found 42.5% scored above the competence scores immediately after training. Similarly, another study compared supported training (assisted by a trainer) and independent web-based CBT training of 8–9 h for eating disorders. No significant differences were found between both groups at post-training and almost half (48%) therapists met the threshold of competence at 6 months post-training (Cooper *et al*. 2017). In Brazil, Pereira *et al*. (2015*a*) evaluated a web-based program to educate primary school teachers about childhood mental disorders and found that teachers in the web-based program had greater improvement in knowledge and understanding about mental disorders as compared to control groups. A pre-post study evaluated of an online course to enhance health professionals' knowledge about the clinical management of alcohol misuse in Brazil demonstrated significant improvement in knowledge about the clinical management of alcohol-related problems (Pereira *et al*. 2015*b*). Hamdani *et al*. (2015) tested the effectiveness of training lay individuals (volunteer family members of children with developmental disorders) in behavioral management skills in rural Pakistan, and found technology-assisted training feasible and effective in improving outcomes of children with developmental disorders. Our findings are consistent with and add to this growing evidence in support of technological enhancements to training for mental health interventions.

Most studies have used online platforms for training health workers. One limitation of this approach is the requirement of a stable Internet connection that may not be available in remote, rural, resource-poor settings particularly in conflict-affected settings. TACTS employs an offline tablet-based application that can enhance the feasibility of this approach. Other risks of over-reliance on technology include the loss of human social contact, invasion of privacy and confidentiality, coercion or discrimination through tracking of individuals with mental health conditions (Patel *et al*. 2018). Sound policies to regulate the use of technologies, as well as making these widely available even to the most marginalized communities, can circumvent these issues.

Two-thirds of the world's population now owns a mobile phone, half of which are smart phones. Mobile phones also contribute to half of the global Internet traffic. Even in many LMICs in south Asia, Africa, and Central America, mobile phone subscriptions exceed 80% of the population. Internet access is also increasing but varies from region to region, ranging from 34% in Africa to 80% in Europe. Reports indicate that there is an annual 4% increase in mobile phones subscriptions and 7% increase in Internet usage globally. This huge penetration of digital technology, even in the world's most impoverished areas, provide great opportunities to harness the power of the technology to overcome barriers and bridge the treatment gap for mental health problems. As technology becomes cheaper and more accessible, such approaches can be further refined so that immediate care is made accessible to prevent the sequelae of traumatic stress, anxiety, and depression as such communities rebuild.

This study has some limitations. It was conducted in a small but hard to reach area of conflict-affected Pakistan. We were unable to follow 25% of the sample at 3 months follow-up. However, we anticipated this keeping in view the context and accounted for this attrition in sample calculations. Longer term evaluation of LHWs' competencies was not carried out to assess the ability of TACTS in maintaining their levels of competency. Critically, our study did not evaluate the outcomes of intervention delivery to the target population. Future studies in larger populations, using a variety of health care providers and measuring clinical outcomes in patients, can furnish further evidence about the generalizability and effectiveness of the training.

## Conclusion

This study suggests that technology can be successfully used to train health workers in hard to reach areas such as post-conflict settings. Scalability of evidence-based interventions in such areas is not feasible with the conventional intense specialist-led face-to-face training and supervision model. Technology-assisted training by non-specialists is equally effective and less costly than the conventional methods of training and supervision. Hence, technology can be a feasible, scalable, cost-effective, and sustainable strategy to train and supervise lay health workers in low-resource settings.

## Footnotes

1 All costs were calculated in Pakistani Rupees; exchange rate PKR110.65 = US$1 (http://www.forex.pk/open-rates.php dated 31 December 2017).

† The notes appear after the main text.
